# Predicting Potential Distribution of the Acanthopanax Sessiliflorus in China Under Future Climate Scenarios Based the Optimized Maximum Entropy (MaxEnt) Model

**DOI:** 10.1002/ece3.73830

**Published:** 2026-06-10

**Authors:** Yang Nan, Shuoning Zhang, Wenjing Xu, Kaize Feng, Ziyang Liu, Qingfeng Wang, Xu Fan

**Affiliations:** ^1^ Organization Liaoning University of Traditional Chinese Medicine Shenyang China

**Keywords:** *Acanthopanax sessiliflorus* (Rupr. Maxim.) Seem., future emission scenarios, habitat, Maximum Entropy (MaxEnt), temperate ecosystems

## Abstract

*Acanthopanax sessiliflorus* (Rupr. Maxim.) Seem. is an ecologically and medicinally important shrub in East Asian temperate forests, yet its habitat dynamics under climate change remain poorly quantified. In this study, an optimized Maximum Entropy (MaxEnt) model was developed using climatic, soil, and topographic variables to project habitat suitability under four future climate scenarios (SSP126, SSP245, SSP370, SSP585) from the 2040s to the 2100s. Model performance was high, with a mean training AUC of 0.9735 and a testing AUC of 0.9660. The current suitable habitat covers 89.97 × 10^4^ km^2^, with a centroid at 118.800934° E, 39.230015° N. Highly suitable areas (3.05 × 10^4^ km^2^) are concentrated in Hebei, Beijing, Liaoning, and the Changbai Mountains. Temperature seasonality (Bio4, 41.5%) was identified as a dominant factor, while maximum temperature of the warmest month (Bio5) contributed important independent information. Future projections show strong scenario dependence. Based on projections for the 2100 time point, highly suitable habitat is predicted to decline across all scenarios: decreasing by 38.43% under SSP126, 62.15% under SSP245, 70.20% under SSP370, and 79.27% under SSP585. Total suitable habitat is projected to increase by 29.74% under SSP126, due to the expansion of marginally suitable areas. In contrast, SSP245, SSP370, and SSP585 show initial expansion followed by long‐term contraction, with a 21.74% net loss under SSP585 by 2100. Habitat centroids are projected to shift northwestward, indicating spatial redistribution. Overall, intensive future emission scenarios will degrade core habitats and reorganize these temperate ecosystems. By identifying climatic sensitivities, this study provides a scientific foundation for optimizing cultivation, managing wild resources, and developing climate‐adaptive conservation strategies to ensure the sustainable utilization of this medicinal shrub.

## Graphical abstract

In this study, we employed an optimized MaxEnt model combined with environmental variables to predict the potential habitat suitability for Acanthopanax sessiliflorus under various future climate projections. Our findings indicate that climate change will likely alter the geographic distribution of this species, with potential shifts in suitable habitats. These results provide essential insights for conservation strategies and sustainable management of Acanthopanax sessiliflorus in China.

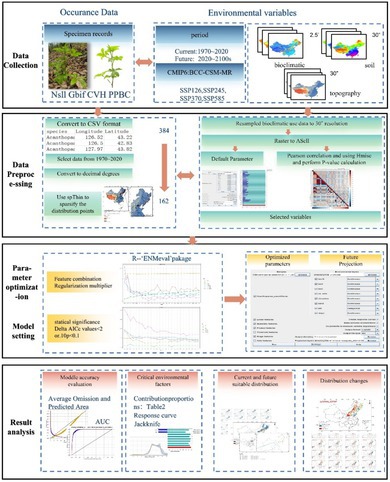

## 1 Introduction

*Acanthopanax sessiliflorus* (Rupr. & Maxim.) Seem. ( *A. sessiliflorus* ), a member of the genus Acanthopanax within the Araliaceae family, is a deciduous shrub widely distributed throughout the temperate forests of East Asia. It primarily occurs in the Changbai Mountains as well as the Hebei, Beijing, and Liaoning regions of China (Flora of China Editorial Committee of Chinese Academy of Sciences 1980). This species plays a crucial role in ecosystem stability by enhancing soil stability and maintaining forest biodiversity (Wang et al. 2016). Additionally, *A. sessiliflorus* has significant medicinal value in traditional medicine, with its rhizomes known for their anti‐inflammatory, antioxidant, and immunomodulatory properties. Previous studies have shown that this plant is rich in saponins, flavonoids, and polyphenols, which exhibit pharmacological activities such as anti‐aging effects and neuroprotective benefits (Liu et al. 2024). As both a medicinal and edible plant (Sun et al. 2023), *A. sessiliflorus* has considerable economic value. Greenhousee cultivation of *A. sessiliflorus* for vegetable production typically yields 900–1100 kg per 667 m^2^ with an income of 40,000–43,000 RMB; meanwhile, cold‐frame greenhouse production yields 800–1000 kg per 667 m^2^ with an income of 20,000–22,000 RMB (Wang and Wang 2023). However, increasing market demand and excessive harvesting have led to a decline in wild populations of *A. sessiliflorus* , raising growing concerns regarding its conservation (Ren 2022).

In addition to anthropogenic exploitation, climate change has emerged as a major long‐term threat to the persistence of *A. sessiliflorus* . Rising temperatures and altered precipitation regimes are reshaping environmental suitability patterns across temperate East Asia. These environmental changes can directly affect the physiological tolerance limits of plants and indirectly modify interspecific competition and habitat quality. Previous studies have suggested that warming trends may drive suitable habitats for temperate woody species to shift toward higher latitudes and elevations (Kim et al. 2011). *A. sessiliflorus* , whose distribution is closely associated with cool‐temperate and montane environments, these shifts may lead to habitat contraction in southern or low‐altitude regions and increased fragmentation of suitable areas. In subalpine zones of Northeast China and the Korean Peninsula, climatic constraints have already been reported to restrict its habitat continuity and regeneration potential (You and Kwon 2015). These combined pressures highlight the urgency of quantitatively assessing how environmental factors determine the species' current distribution and how future climate scenarios may reshape its potential range.

To address this need for quantitative assessment, species distribution models (SDMs) integrate species occurrence points with environmental data to predict potential habitat suitability an important tool for ecological and conservation research. Common SDM methods include statistical models such as generalized linear models (GLMs) and generalized additive models (GAMs) (Wang et al. 2025), which are sensitive to missing data; machine learning models, such as random forests (Hanberry 2024) which are often computationally complex and function as “black box” models (Dong 2024); and overly simplified niche models, such as Bioclim (Serrano‐Notivoli et al. 2022). Given the limitations of these models in robustness, interpretability, and computational efficiency, the Maximum Entropy (MaxEnt) model was selected for this study as it effectively balances prediction accuracy, the ability to handle complex nonlinear relationships, and tolerance to incomplete data.

Among SDMs, the Maximum Entropy (MaxEnt) model is widely applied due to its suitability for presence‐only data (Phillips et al. 2006). Compared with other methods, MaxEnt performs well even with limited or spatially biased data. However, the efficacy of the model is contingent upon the quality and spatial structure of the input data; biased sampling or data volume can reduce predictive accuracy. Consequently, optimization of the parameters is imperative for enhancing the model's accuracy (Merow et al. 2013). MaxEnt has been successfully applied to habitat suitability predictions for various species, including *Comanthera elegans (Bong.) L.R. Parra & Giul* (de Azevedo et al. 2026), *Hippophae tibetana Schltdl* (Ma et al. 2026), and *Arabidopsis thaliana* (Shi et al. 2023). Given its proven ability to better balance prediction accuracy, handle complex nonlinear relationships, and tolerate incomplete data (Wang, Zhang, and Zhao 2022), the MaxEnt model was selected for this study.

However, despite the demonstrated efficacy of MaxEnt in these studies and the ecological importance of *A. sessiliflorus* , large‐scale quantitative assessments of its potential distribution under future climate change remain limited. In particular, the extent to which different greenhouse gas emission pathways may alter the spatial distribution, habitat quality, and geographic stability of this species has not been systematically evaluated. Therefore, this study employs an optimized MaxEnt model to simulate the habitat suitability of *A. sessiliflorus* from 2021 to 2100 under multiple Shared Socioeconomic Pathways (SSP126, SSP245, SSP370, and SSP585).

Furthermore, this research offers significant practical guidance for the introduction and cultivation of the species; by predicting habitat shifts under various climate scenarios, it provides a rigorous scientific basis for optimizing cultivation sites, managing wild resources, and developing conservation strategies tailored to mitigate the impacts of climate change.

## 2 Materials and Methods

### 2.1 Research Framework

**FIGURE 1:**
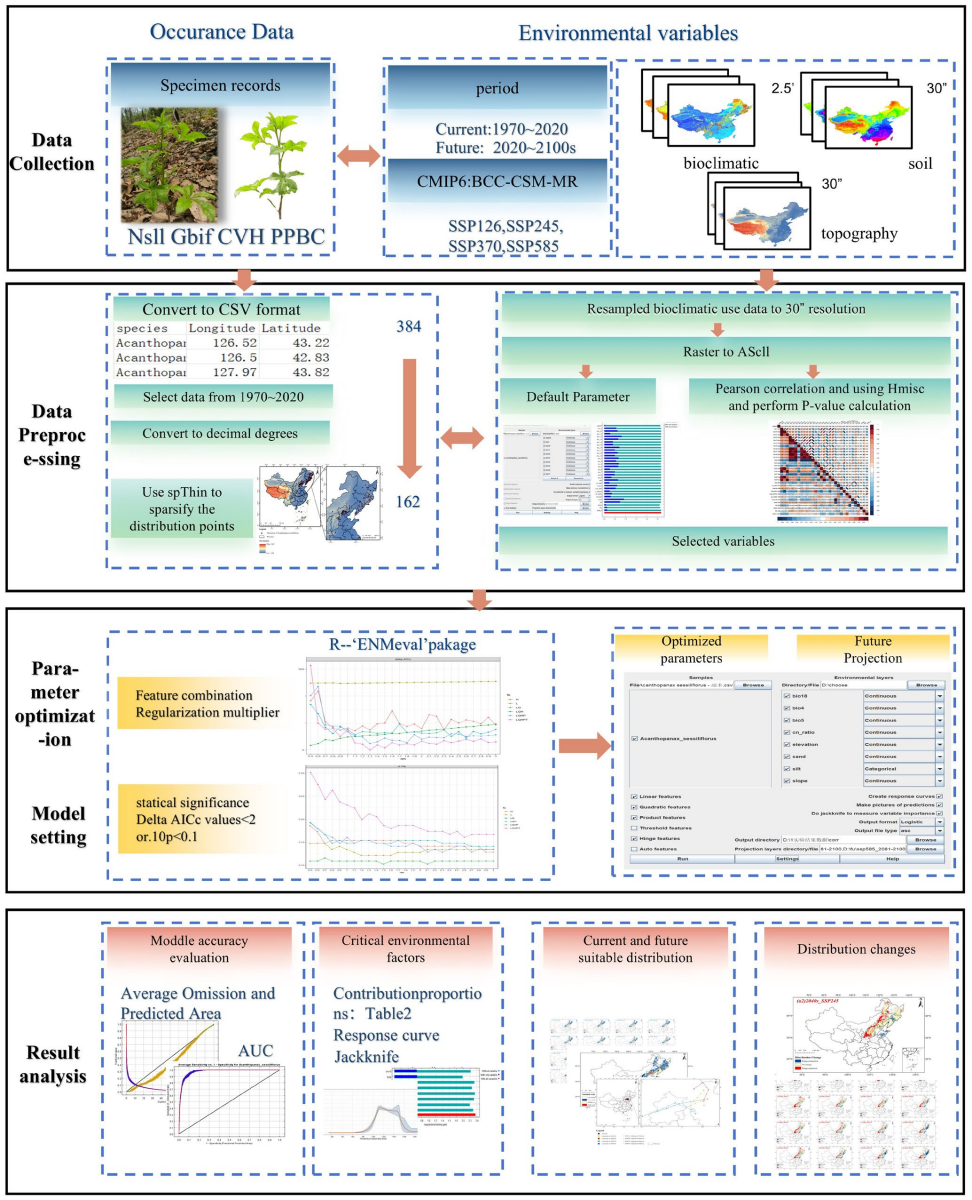
The framework of this study's contribution.

Based on the research objectives and the basic principles of the MaxEnt model, a framework (Figure 1) was constructed to simulate the current and future distribution of *A. sessiliflorus* in the suitable habitat. The framework consists of four main parts: (1) Data collection: the distribution data of the species of *A. sessiliflorus* and the environmental variables, including topographic, bioclimate, and soil factors were collected. (2) Data processing: Species distribution data were organized into .csv files including species name, longitude, latitude. Records outside the study area were filtered using the R package “spThin” to eliminate samples that were close together as well as duplicates. Environmental variables were cropped to the study area, resampled to a spatial resolution of 1 km, and screened for multicollinearity using Pearson correlation analysis, with highly correlated variables excluded. (3) Parameter optimization: The “ENMeval” package in R was utilized to select optimal parameters, combining Feature Combination (FC) and Regularization Factor (RM). The MaxEnt model for *A. sessiliflorus* was then constructed and projected to future periods. (4) Simulation and evaluation: The accuracy of the model was evaluated based on the Area Under the Curve (AUC). Dey environmental factors were identified based on percent contribution, permutation importance, response curves and jackknife tests. Finally, spatiotemporal changes and evolutionary trends in suitable habitats of *A. sessiliflorus* under current and future climate scenarios were simulated and analyzed.

### 2.2 Species Occurrence Data

**FIGURE 2:**
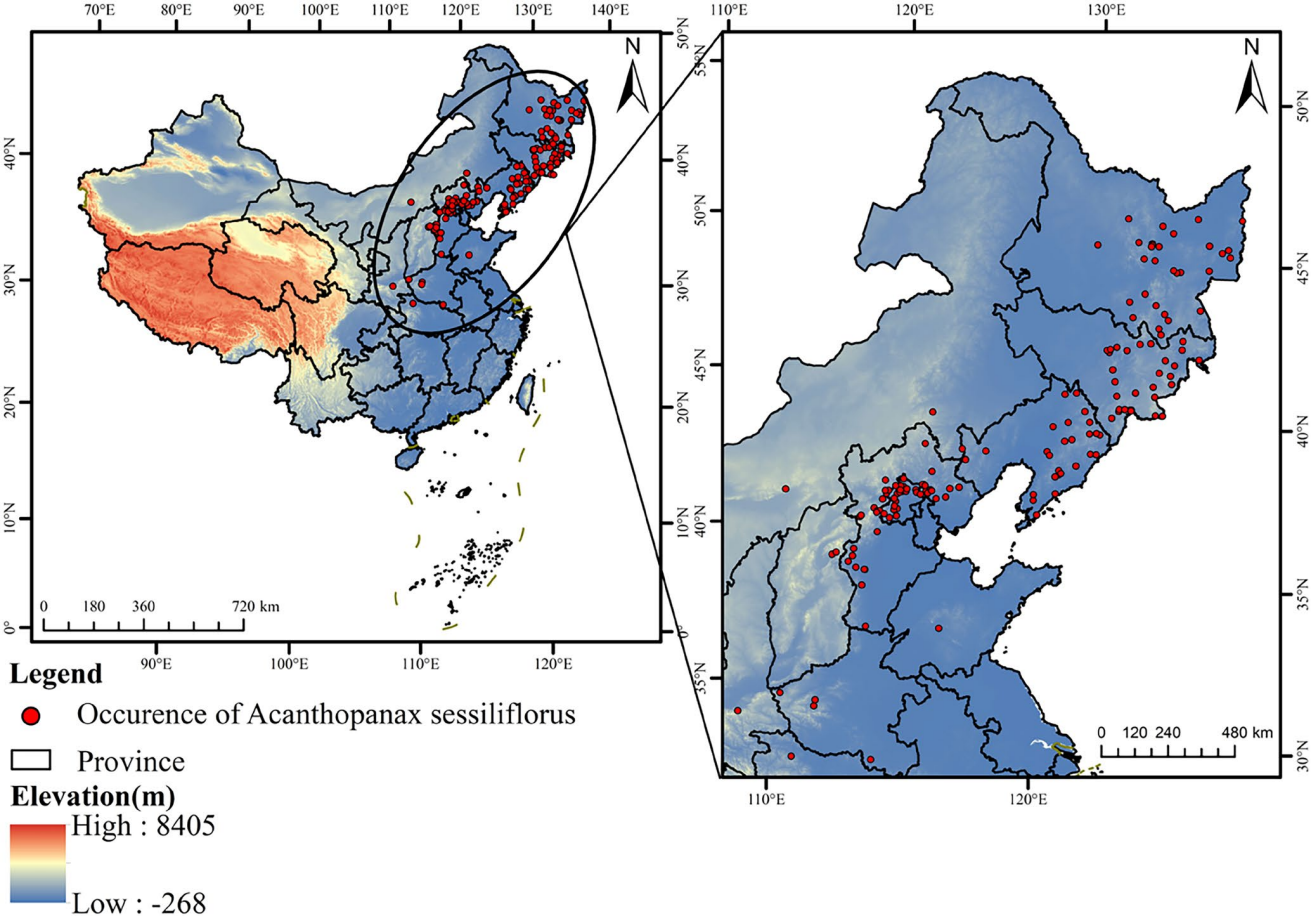
Distribution of *A. sessiliflorus* species occurrence points.

In this study, occurrence data of *A. sessiliflorus* were collected from the following databases: the National Specimen Information Infrastructure (NSII, http://www.nsii.org.cn/), the Global Biodiversity Information Facility (GBIF, https://www.gbif.org/), the Chinese Virtual Herbarium (CVH, https://www.cvh.ac.cn), and the Chinese Plant Photo Bank (PPBC, http://ppbc.iplant.cn/). The preliminary dataset yielded by this process contains 384 occurrence records. It is imperative to ensure that the sampling time of occurrence data is consistent with the time range of environmental variables. To prevent sampling bias and overfitting caused by the small spacing between distribution points, the “spThin” package (Aiello‐Lammens et al. 2015) was used to sparse the data. This process ensured that only one distribution point was retained within a 1 km radius. In the end, 162 valid distribution points were obtained (Figure 2) and formatted as a .csv file containing the species name, longitude and latitude for the subsequent MaxEnt modeling.

### 2.3 Environmental Variables

This study selected 32 environmental variables, including 19 bioclimatic factors, 10 soil factors, and 3 topographic factors (Table 1), to construct a species distribution model (SDM) for *A. sessiliflorus* . Bioclimatic data were obtained from the WorldClim database (https://worldclim.org/), with current climate data derived from historical climate observations and future climate data simulated based on the Coupled Model Intercomparison Project Phase 6 (CMIP6) (O'Neill et al. 2016). The future climate projections were generated using the Beijing Climate Center Climate System Model 2 Medium Resolution (BCC‐CSM2‐MR) (Wu et al. 2021) under four Shared Socioeconomic Pathways (SSPs): SSP126, SSP245, SSP370, and SSP585 to represent low, medium, high, and very high greenhouse gas emission scenarios, respectively. These projections covered four future periods: 2021–2040, 2041–2060, 2061–2080, and 2081–2100, at a spatial resolution of 2.5′ (5 km × 5 km). Soil data were obtained from the Harmonized World Soil Database (HWSD, https://www.fao.org/), with a spatial resolution of 30′′ (1 km × 1 km). Topographic factors included elevation, slope, and aspect, which were derived from elevation data provided by the China Resource and Environmental Science and Data Platform (https://www.resdc.cn/). Slope and aspect were extracted using ArcGIS, and all variables were processed at a spatial resolution of 30′′ (1 km × 1 km). Notably, these variables were assumed to remain constant across all future climate scenarios because no projected future layers are available for HWSD soil or topographic variables (Liang et al. 2021).

**TABLE 1:** Variables used in the model prediction.

Variable description	Abbreviated name	Unit
bio1	Annual mean temperature	°C
bio2	Mean diurnal range (mean of monthly (max temp–min temp))	°C
bio3	Isothermality (BIO2/BIO7) (×100)	—
bio4	Temperature seasonality (standard deviation ×100)	°C
bio5	Max temperature of warmest month	°C
bio6	Min temperature of coldest month	°C
bio7	Temperature annual range (BIO5–BIO6)	°C
bio8	Mean temperature of wettest quarter	°C
bio9	Mean temperature of driest quarter	°C
bio10	Mean temperature of warmest quarter	°C
bio11	Mean temperature of coldest quarter	°C
bio12	Annual precipitation	mm
bio13	Precipitation of wettest month	mm
bio14	Precipitation of driest month	mm
bio15	Precipitation seasonality (coefficient of variation)	—
bio16	Precipitation of wettest quarter	mm
bio17	Precipitation of driest quarter	mm
bio18	Precipitation of warmest quarter	mm
bio19	Precipitation of coldest quarter	mm
clay	Clay	—
cn_ratio	Carbon nitrogen ratio (C/N)	C: N
coarse	Coarse fragments	—
org_carbon	Organic carbon content	—
ph_water	pH in water	—
ref_bulk	Reference bulk density	g/cm^3^
sand	Sand	—
silt	Silt	—
teb	Teb	cmolc/kg
total_n	Total nitrogen content	g/kg
elevation	Elevation	m
slope	Slope	°
aspect	Aspect	—

*Note:* “—” denotes no units.

All environmental variables were processed using the terra package in R (Hijmans 2025). The layers were resampled to a uniform spatial resolution of 30″ (approximately 1 km × 1 km) and projected to the GCS_Krasovsky_1940 coordinate system. To reduce multicollinearity among environmental variables, which can lead to model overfitting and reduced predictive generalization. Pearson correlation coefficients and significance levels (*p* < 0.05) were calculated for the 32 variables using the rcorr function in the Hmisc package (Harrell Jr and Dupont 2025). In cases where a pair of variables exhibited a high correlation (|*r*| ≥ 0.8), only one variable from the pair was retained for subsequent modeling. Furthermore, we conducted jackknife analyses and examined percent contribution and permutation importance from preliminary MaxEnt runs to further refine our variable set, retaining only those with high independent explanatory power and clear ecological relevance.

### 2.4 Optimization and Model Parameters

The MaxEnt model is grounded in the maximum entropy principle derived from statistical mechanics (Jaynes 1957). A key strength of this model lies in its ability to accurately assess environmental suitability using only species presence data (presence‐only data), making it extensively applied in species distribution prediction (Tümer et al. 2026). By integrating species occurrence records with environmental factors (e.g., temperature, precipitation, topography), the model quantitatively characterizes spatial patterns of habitat suitability (Phillips and Dudík 2008). Mathematically, MaxEnt employs constrained optimization algorithms for parameter estimation (Korbel 2021), with its probability distribution expressed as:

(1) $$P\left(x\right)=\frac{1}{z}\exp\left(\sum_{i=1}^{n}\lambda_{i}f_{i}\left(x\right)\right)$$

In this study, $P\left(x\right)$ represents the conditional probability of species presence given environmental variables, $f_{i}\left(x\right)$ denotes feature functions that describe the relationship between environmental factors and species distribution, *λ*_*i*_ represents the corresponding feature weights, and Z is the normalization factor ensuring probability completeness. The model employs the Lagrange multiplier method to solve the constrained optimization problem, ensuring that the predicted feature expectations match the empirical expectations from training data (Phillips et al. 2017).

MaxEnt model performance is highly dependent on two key hyperparameters: Feature Classes (FC) and the Regularization Multiplier (RM). FC determines the mathematical form of feature functions $f_{i}\left(x\right)$, including linear (L), quadratic (Q), product (P), and threshold (H) features (Morales et al. 2017). Quadratic terms capture nonlinear environmental effects, while product terms represent interactive relationships among variables. RM controls the magnitude of weight coefficients *λ*_*i*_ via L1 or L2 regularization, balancing model complexity and generalization ability (Radosavljevic and Anderson 2014). Higher RM values enhance regularization strength, reducing overfitting risks but potentially omitting weak signals. Studies have shown that optimizing FC and RM via cross‐validation significantly improves model robustness to noise while preserving ecological interpretability (Merow et al. 2013).

In this study, we optimized FC and RM using the “ENMeval” package (https://github.com/jamiemkass/ENMeval) in R version 4.4.2 (https://cran.r‐project.org/). The optimization process set RM values from 0.5 to 3 in increments of 0.1 and evaluated five FC combinations: “L”, “LQ”, “H”, “LQH”, and “LQHP” using cross‐validation (Zhao, Wang, and Chen 2024). The models were selected according to these criteria (Elith et al. 2011): (1) excluding models with low AUC (auc.val < 0.7); (2) removing overfitted models (auc.diff > 0.1); (3) eliminating models with high omission rates (or.10p > 0.1); (4) selecting only models with delta.aicc < 2. The final optimal model was selected based on high AUC and moderate auc.diff while ensuring a low omission rate. When delta.aicc differences were minimal, the model with the highest w.aicc but simpler structure was preferred. Based on this process, the optimal parameter combination was determined to be FC = LQHP and RM = 1.2.

### 2.5 MaxEnt Model

In this study, MaxEnt version 3.4.1 (https://biodiversityinformatics.amnh.org/open_source/MaxEnt/) was used to model species distribution. The model was projected onto the future climate scenarios of the 2040s, 2060s, 2080s, and 2100s, including SSP126, SSP245, SSP370, and SSP585. Based on the optimized FC and RM parameters, the species distribution data were randomly split, with 25% of the data used as the test set and 75% as the training set. The parameters set by the MaxEnt model were “Do jackknife to measure variable importance,” “Random seed,” “a maximum of five thousand iterations,” “Write plot data,” “Create response curves,” “Write background predictions,” “Bootstrap,” “Replicates ten” and “Output format logistic.” The amalgamation of the results from the percent contribution, the permutation importance, and the correlation matrix resulted in the identification of a final set of eight environmental variables.

In the final model construction, the eight retained environmental variables retained from the previous steps were used, while all parameter settings were kept unchanged. The optimized parameter combination was reentered into the MaxEnt model for analysis. Model performance was evaluated using the Receiver Operating Characteristic (ROC) curve, which plots the false positive rate (1 − specificity) on the *x*‐axis against the true positive rate (sensitivity) on the *y*‐axis. The area under the ROC curve (AUC) was used as an index of model goodness‐of‐fit, with values ranging from 0.5 to 1. Values closer to 1 indicate stronger predictive accuracy (Yan et al. 2024). Model accuracy was classified as 0.50–0.60 (fail), 0.61–0.70 (poor), 0.71–0.80 (fair), 0.81–0.90 (good), and 0.91–1.0 (excellent).

### 2.6 Potentially Habitat Suitability Partitions

Based on ASC data output from the MaxEnt model, spatial analysis was performed in GIS. First, the ASC file was converted into a raster format and overlaid on the administrative division map of China for visualization analysis, and then the habitat suitability was reclassified using GIS. Habitat suitability values were then reclassified using GIS tools.

Specifically, the mean threshold value of the “Maximum Sensitivity Plus Specificity” method was calculated based on ten replicate experiments. This threshold selection method balances the sensitivity and specificity of the training dataset, effectively reducing false positive and false negative errors (Liu et al. 2013). Based on this mean threshold, the study area was initially classified into suitable and unsuitable habitat. Furthermore, following the recommendations of the IPCC (Sun et al. 2012) and considering the biological characteristics of *A. sessiliflorus* , suitable habitats were further categorized into four levels: high‐suitability habitat (0.66 ≤ *p* < 1), moderate‐suitability habitat (0.33 ≤ *p* < 0.66), low‐suitability habitat (0.1374 ≤ *p* < 0.33), and unsuitable habitat (0.1 ≤ *p* < 0.1374).

In the spatial statistical analysis phase, the raster calculation tool was used to quantify the area corresponding to each habitat suitability level. Additionally, based on the SDM toolbox in ArcGIS, the centroid coordinates of the species distribution at different periods were computed to construct a centroid migration trajectory model. This model helps to reveal the spatiotemporal evolution of suitable habitat for *A. sessiliflorus* , providing a scientific basis for future species conservation and management.

## 3 Result

### 3.1 Model Accuracy and the Current Distribution

The MaxEnt model was employed to perform habitat suitability modeling, with ten repetitions conducted to evaluate model stability. The average training AUC was found to be 0.9735 (standard deviation = 0.002), while the average testing AUC was 0.9660 (standard deviation = 0.0116). These results indicate that the *A. sessiliflorus* habitat suitability prediction model exhibits high stability and reliability. Previous studies have suggested that models with AUC greater than 0.9 are considered as “excellent,” demonstrating the robustness of our model in evaluating suitable habitat for this species.

**FIGURE 3:**
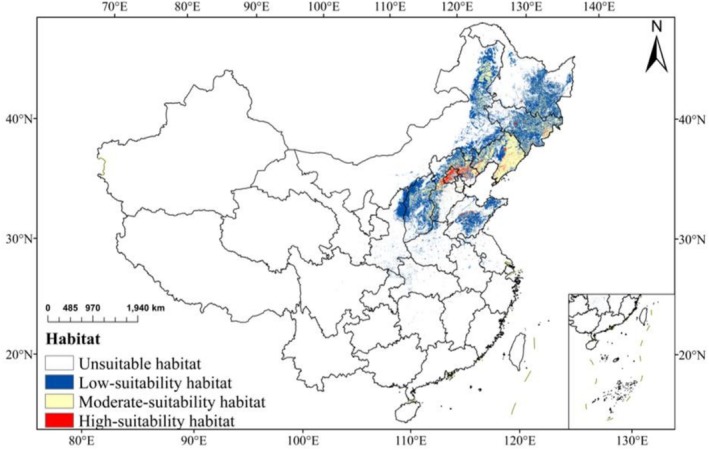
The potential suitable habitat of *A. sessiliflorus* species in China.

Based on the predicted habitat suitability distribution, the suitable habitat of *A. sessiliflorus* was classified into four levels (Figure 3). Using the average “Maximum Sensitivity Plus Specificity” threshold from ten model runs, areas with an occurrence probability of ≥ 13.74% were designated as suitable habitat. The suitable habitat was calculated to be 89.9697 × 10^4^ km^2^. Specifically, the high‐suitability habitat covered 3.04546 × 10^4^ km^2^, the moderate‐suitability habitat covered 29.8291 × 10^4^ km^2^, and the low‐suitability habitat covered 57.0951 × 10^4^ km^2^. These habitats were mainly distributed in Baoding (Hebei Province), Beijing, most parts of Liaoning Province, and the Changbai Mountain region.

### 3.2 Critical Environmental Factors

Following the initial Pearson correlation screening (Figure S1), the remaining variables were further filtered based on their percent contribution, permutation importance (Table S1), and jackknife test results (Figure S2). This multi‐step process narrowed the initial 32 variables down to eight environmental variables. Among the eight environmental variables used for modeling (Table 2), the most critical factors affecting the habitat suitability of *A. sessiliflorus* were Temperature Seasonality (bio4, percent contribution: 41.5%), Precipitation of Warmest Quarter (bio18, percent contribution: 31.2%), and silt content (percent contribution: 14.4%). The percent contribution and permutation importance of these three factors exceeded 80%, indicating their crucial role in model construction.

**TABLE 2:** The contribution proportions and cumulative proportion contributions of the top 7 environmental variables.

Variable	Percent contribution (%)	Permutation importance (%)
Temperature seasonality (bio4)	41.5	39.3
Precipitation of warmest quarter (bio18)	31.2	40.3
Silt	14.4	4.1
Carbon nitrogen ratio (cn_ratio)	6.3	3.2
Slope	4.6	3.4
Sand	1.1	3.2
Max temperature of warmest month (bio5)	0.7	2.6
Elevation	0.2	3.9

The response curves further illustrate the relationships between environmental variables and the probability of *A. sessiliflorus* occurrence. As bio18 increases, the probability of occurrence first increases rapidly and then decreases slowly. Bio4 exhibits a saddle‐shaped pattern, while silt shows a fluctuating trend, generally following a parabolic shape.

**FIGURE 4:**
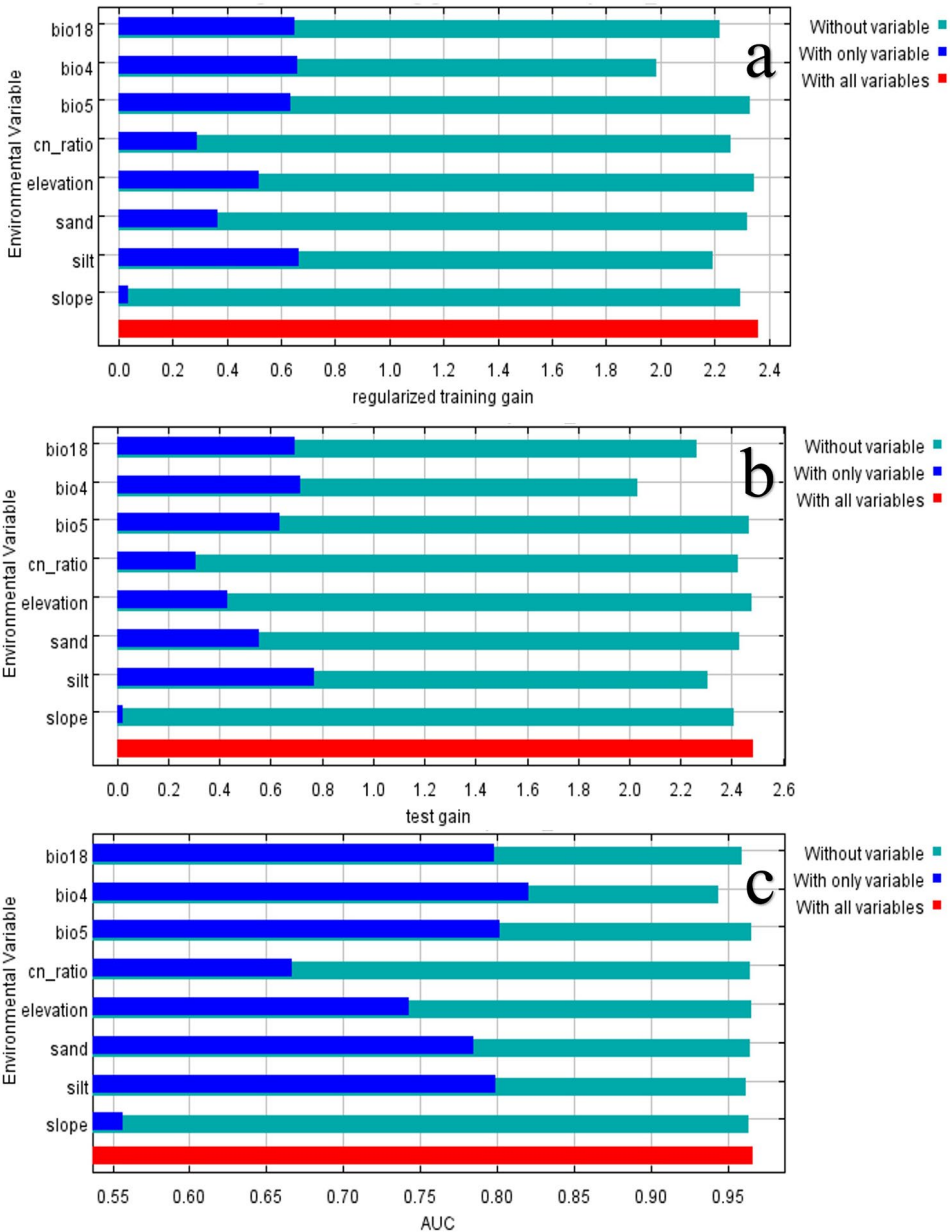
Jackknife test of environmental variables in MaxEnt: (a) Jackknife of training gain for *A. sessiliflorus* ; (b) Jackknife of test gain for *A. sessiliflorus* ; (c) Jackknife of AUC for *A. sessiliflorus* .

Additionally, the Jackknife test result compared training scores under three conditions: “excluding a given variable,” “using only that variable,” and “using all variables.” When only a single variable was retained, those with higher training scores provided greater regularization gain, indicating that they contained unique information not present in other variables (Figure 4). In contrast, variables with higher testing scores contributed more effectively to model construction. The results revealed that bio4 contained a significant amount of information not found in other variables, while silt played a critical role in model performance. Although bio5 had a lower percent contribution and permutation importance in Table 2, it still contained a considerable amount of unique information. When used as the sole predictor, bio5 achieved a testing AUC of 0.801, which was comparable to that of bio18 (0.798). These findings suggest that bio5 should also be considered a critical environmental factor influencing the distribution of *A. sessiliflorus* .

**FIGURE 5:**
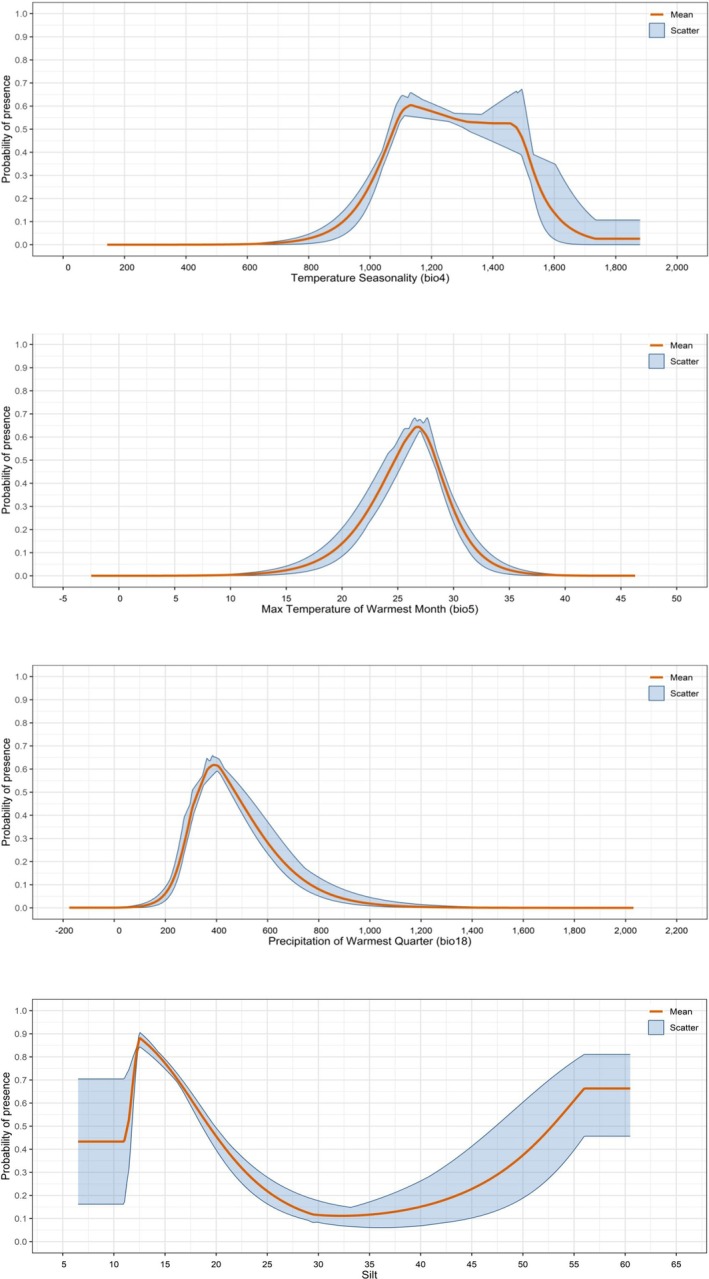
Probability of *A. sessiliflorus* species and environmental factors.

The relationship between the probability of *A. sessiliflorus* occurrence and variable values is illustrated in Figure 5. For continuous variables, the blue‐shaded area represents the range between minimum and maximum occurrence probabilities, while the orange curve indicates the average probability. For the categorical variable silt, the red region represents the mean probability, the blue region denotes the minimum probability, and the orange region shows the maximum probability.

Furthermore, using the probability threshold of the average Maximum Sensitivity Plus Specificity as a criterion, the suitable range and optimal values of key environmental variables were determined (Table 3). The suitable range of bio4 (Temperature Seasonality) was 940.21–1599.91, with an optimal value of 1132.9. The suitable range of bio18 (Precipitation of Warmest Quarter) was 236.80–20.13 mm, with an optimal value of 389.08 mm. For bio5 (Max Temperature of Warmest Month), the suitable range was 20.05°C–31.60°C, with an average value of 26.82°C. The suitable silt content ranged from 6.5% to 28.2% and 38.2% to 60.5%, with an optimal value of 12.5%. In summary, *A. sessiliflorus* is generally found in regions with high temperature seasonality, moderate precipitation, relatively low summer temperatures, and well‐aerated soil.

**TABLE 3:** The threshold values (suitability index > 13.74%) for each critical environmental variable.

Variable	Threshold value (SI > 13.74%)	
	Value range	Maximum SI's value
Temperature seasonality (bio4)	940.21–1599.91	1132.91
Precipitation of warmest quarter (bio18)	236.80–720.13	389.08
Silt	6.5–28.2, 38.2–60.5	12.5
Max temperature of warmest month (bio5)	20.05–31.60	26.82

### 3.3 Potential Distribution of *A. sessiliflorus* Species in Future Climatic Scenarios

**FIGURE 6:**
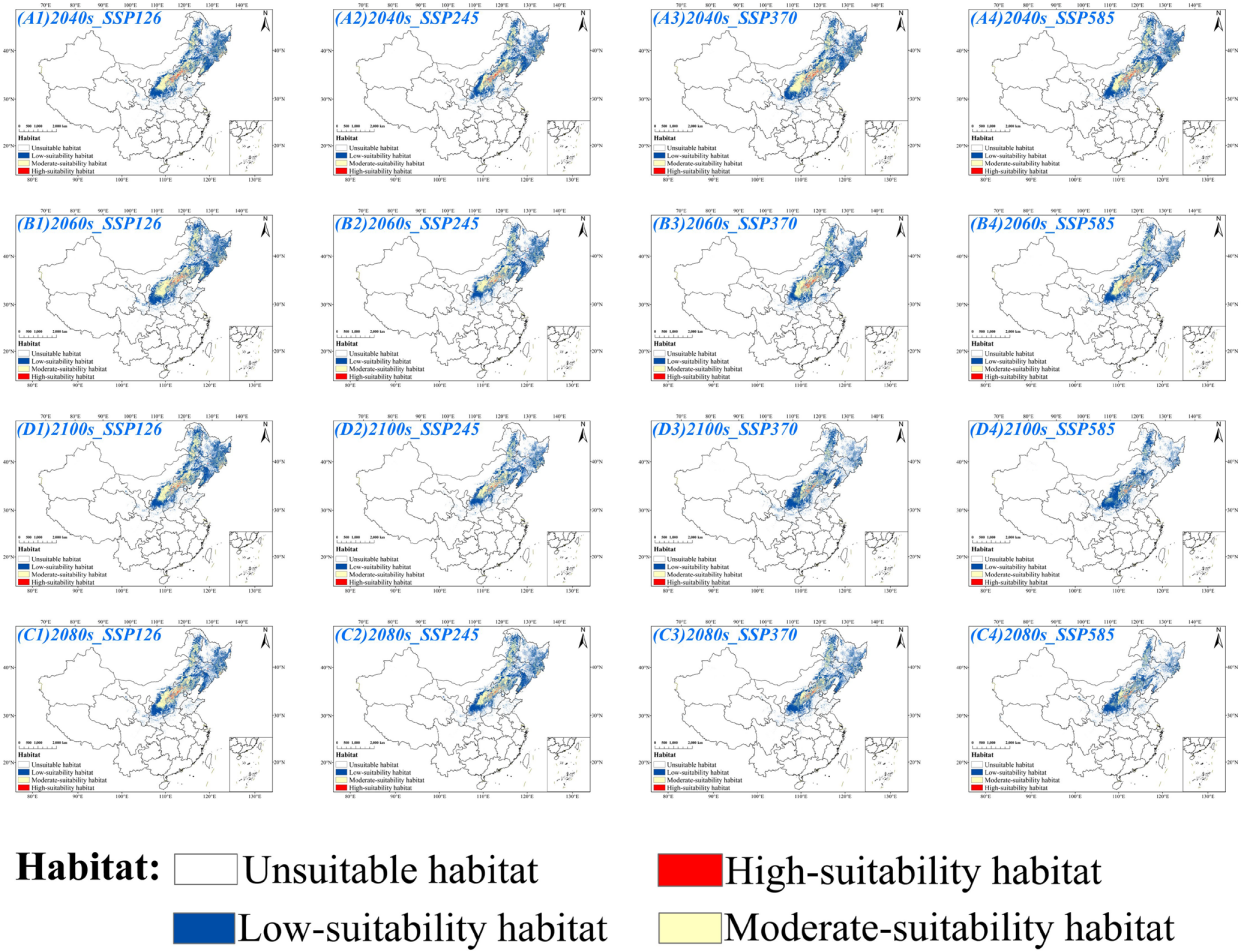
The habitat suitability of *A. sessiliflorus* in China under 4 Shared Socioeconomic Pathways in the years 2040s (A1–A4), 2060s (B1–B4), 2080s (C1–C4), and 2100s (D1–D4).

**FIGURE 7:**
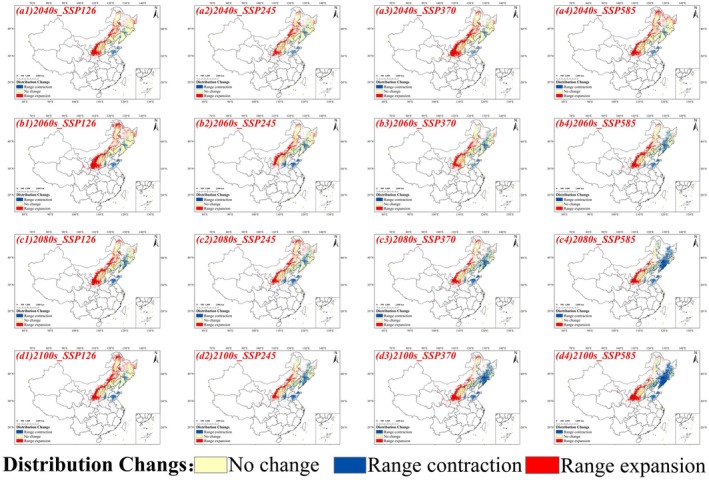
Projected changes in the habitat area of *A. sessiliflorus* in China under four Shared Socioeconomic Pathways (SSPs) during the 2040s (a1–a4), 2060s (b1–b4), 2080s (c1–c4), and 2100s (d1–d4).

As a climate‐sensitive species, *A. sessiliflorus* is likely to experience significant changes in its future distribution due to global warming. To assess the potential impacts of climate change on its habitat suitability, this study simulated the dynamic changes in suitable habitat under four Shared Socioeconomic Pathways (SSPs: SSP126, SSP245, SSP370, and SSP585). Table 4 quantifies the changes in high‐suitability, moderate‐suitability, and low‐suitability habitat, as well as the total suitable habitat (×10^4^ km^2^) and their respective change rates (%) for the 2040s, 2060s, 2080s, and 2100s. Figure 6 visually illustrates the projected spatial distribution of future suitable habitat, categorizing it into high‐suitability, moderate‐suitability, and low‐suitability habitat, as well as unsuitable areas. Additionally, Figure 7 compares the overlap between current and future suitable habitat, revealing three dynamic patterns: contraction zones, expansion zones, and no change zones.

**TABLE 4:** Changes in suitability habitats for *A. sessiliflorus* species over time under different SSP scenarios.

Acanthopana× sessiliflorus	Period	High‐suitability habitat (×10^4^ km^2^)	Change (%)	Moderate‐suitability habitat (×10^4^ km^2^)	Change (%)	Low‐suitability habitat (×10^4^ km^2^)	Change (%)	Suitability habitat (×10^4^ km^2^)	Change (%)
	Current	3.04546		29.8291		57.0951		89.96966	
SSP126	2040s	2.46063	−19.20	38.2681	28.29	68.3588	19.73	109.08753	21.25
	2060s	2.28741	−24.89	41.0529	37.63	77.3139	35.41	120.65421	34.11
	2080s	2.08534	−31.53	35.4722	18.92	69.5156	21.75	107.07314	19.01
	2100s	1.87508	−38.43	38.4684	28.96	76.3864	33.79	116.72988	29.74
SSP245	2040s	2.38146	−21.80	33.8081	13.34	71.8708	25.88	108.06036	20.11
	2060s	1.446816	−52.49	29.989528	0.54	63.5349	11.28	94.971244	5.56
	2080s	1.66293	−45.40	29.943	0.38	68.5296	20.03	100.13553	11.30
	2100s	1.15284	−62.15	24.3729	−18.29	57.3999	0.53	82.92564	−7.83
SSP370	2040s	2.57249	−15.53	43.0627	44.36	72.5943	27.15	118.22949	31.41
	2060s	2.17867	−28.46	32.8945	10.28	62.8831	10.14	97.95627	8.88
	2080s	1.26379	−58.50	23.504	−21.20	64.5367	13.03	89.30449	−0.74
	2100s	0.907543	−70.20	18.1604	−39.12	63.2165	10.72	82.284443	−8.54
SSP585	2040s	2.51899	−17.29	37.5581	25.91	74.2874	30.11	114.36449	27.11
	2060s	1.54756	−49.18	29.3174	−1.72	61.8858	8.39	92.75076	3.09
	2080s	0.808618	−73.45	16.452	−44.85	53.7236	−5.91	70.984218	−21.10
	2100s	0.631244	−79.27	12.1068	−59.41	57.6718	1.01	70.409844	−21.74

The results show significant differences in suitable habitat changes under different SSP scenarios. Overall, high‐emission scenarios accelerate habitat degradation, whereas low‐emission scenarios provide a degree of buffering. Across all scenarios, the high‐suitability habitat shows a marked decline over time, with loss rates increasing with emission intensity. By the 2100s, the high‐suitability habitat is projected to shrink by −38.43% under SSP126 and by −79.27% under SSP585. The moderate‐suitability habitat exhibits scenario‐dependent variations: it expands under SSP126 (+28.96%) but generally declines in other scenarios, with SSP585 showing the most extreme reduction (−59.41%). The low‐suitability habitat fluctuates significantly, decreasing in most scenarios except SSP126. Notably, SSP370 and SSP585 exhibit negative growth in the later periods. Regarding total suitable habitat, only SSP126 shows an increase (+29.74%), whereas all other scenarios indicate a decline, with SSP585 experiencing the largest decrease (−21.74%).

A closer examination of habitat changes under different scenarios reveals distinct patterns. Under SSP126, the expansion of moderate‐suitability and low‐suitability habitats leads to an overall increase in suitable habitat, suggesting increased low‐suitability habitat fragmentation and a potential for species migration to maintain viability. However, under SSP245 and higher‐emission scenarios, habitat quality deteriorates systematically. For instance, under SSP245, the high‐suitability habitat declines by 62.15%, the moderate‐suitability habitat shifts from expansion to reduction in later periods, and total suitable habitat follows a downward trend, indicating that even moderate emissions can negatively impact habitat quality. Under SSP370, the high‐suitability and moderate‐suitability habitats decrease by 70.20% and 39.12%, respectively, while the low‐suitability habitat slows its expansion, leading to both quantitative and qualitative habitat degradation. The SSP585 scenario shows the most severe impact, with high‐suitability habitats decreasing by nearly 80%, moderate‐suitability habitats shrinking by 59.41%, and the suitable habitat continuously declining, underscoring the severe consequences of extreme emissions.

In summary, as emission levels rise, both high‐suitability and moderate‐suitability habitats of *A. sessiliflorus* exhibit a significant decline, while fluctuations in low‐suitability and totally suitable habitats increase. The low‐emission pathway (SSP126) offers a potential buffer for species adaptation, whereas high‐emission pathways (SSP370 and SSP585) exacerbate irreversible habitat degradation. These findings highlight the critical importance of global greenhouse gas mitigation efforts in biodiversity conservation.

### 3.4 Centroid Shifts of *A. sessiliflorus* Species in Future Climatic Scenario

**FIGURE 8:**
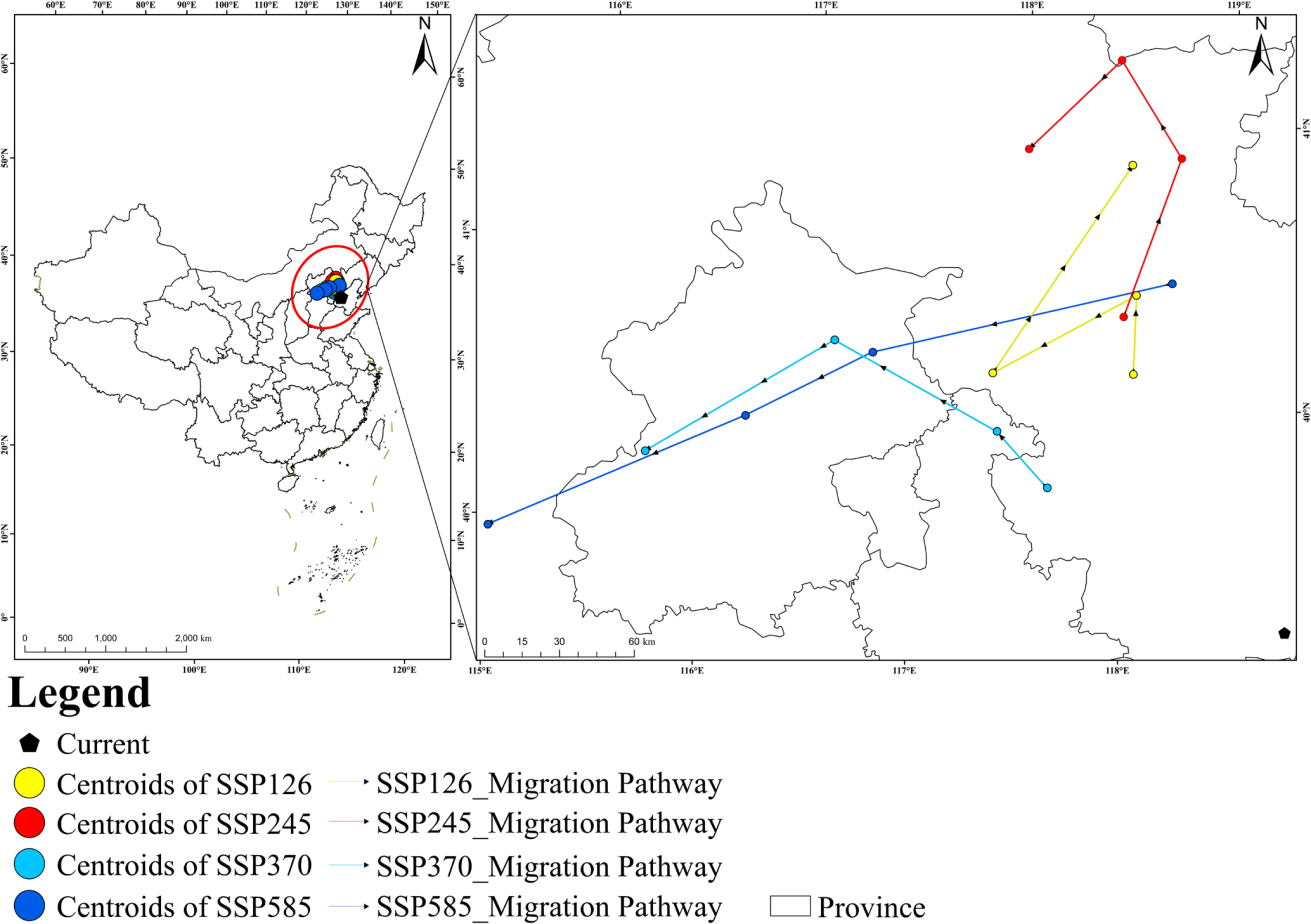
Centroids shifts of *A. sessiliflorus* under future climate scenarios.

The centroid migration trajectory of the future suitable habitat for *A. sessiliflorus* , calculated using ArcGIS (Figure 8), indicates an overall northwestward shift. Under current climatic conditions, the centroid is located in Qianxi County, Tangshan City (118.800934° E, 39.230015° N). Future projections indicate that both the direction and magnitude of centroid migration are strongly associated with emission intensity, with progressively greater westward displacement under higher‐emission scenarios.

Specifically, under the SSP126 (low‐emission) scenario, the centroid migration exhibits periodic fluctuations: shifting northeast from 2021 to 2060, briefly deviating southwest from 2060 to 2080, finally reaching Chengde City (118.3871° E, 40.951333° N) by 2100. Under the SSP245 (moderate‐emission) scenario, the centroid initially migrates northeast toward southwestern Chifeng City, followed by a southwestward reversal after 2080, eventually stabilizing in northern Chengde City (117.898962° E, 41.057088° N). In the SSP370 (high‐emission) scenario, the centroid migrates northwest from 2021 to 2080 before shifting southwest, eventually reaching Yanqing District, Beijing (115.885087° E, 40.154304° N) by 2100. In contrast, under the extreme‐emission SSP585 scenario, the centroid follows a unidirectional southwest trajectory, crossing Beijing and ultimately settling in Zhangjiakou City (115.09659° E, 39.953862° N).

Overall, the centroid migration trajectory of *A. sessiliflorus* is strongly correlated with emission intensity. Low‐emission scenarios result in relatively smaller migration distances and some degree of reversibility, whereas high‐emission scenarios lead to greater migration distances and a more unidirectional shift. In particular, the westward shift of the centroid (a longitudinal shift of −3.7° from SSP126 to SSP585) suggests that climate warming may force the species to contract toward higher latitudes or more topographically complex northwestern inland regions, where there is a more balance between temperature and precipitation suitability.

## 4 Discussion

### 4.1 Effects of the Critical Environmental Variables on the Distribution of *A. sessiliflorus* Species

The distribution and growth of *A. sessiliflorus* are significantly affected by critical environmental factors. In this study, the MaxEnt model was used to determine the dominant variables and their optimal thresholds for its suitable habitat. Temperature Seasonality (bio4, 1132.91), which reflects the annual temperature fluctuation range, plays a crucial role in its ecological distribution. A higher temperature seasonality indicates that this species is primarily distributed in temperate monsoon climate zones, where cold winters and hot summers shape its adaptive strategies. Such seasonal variations may enhance the plant's ability to regulate dormancy and growth, thereby optimizing its physiological responses to climatic stress (Loreto and Atzori 2024). Previous studies have demonstrated that moderate temperature seasonality can drive the programmed regulation of seasonal growth potential and dormancy depth in plants. This process optimizes the trade‐off between growth and stress survival, thereby effectively enhancing the tolerance of temperate woody plants to dehydration stress induced by extreme temperatures (Volaire et al. 2023).

Precipitation of Warmest Quarter (bio18, 389.08 mm) indicates that *A. sessiliflorus* primarily grows in regions where summer precipitation is concentrated, closely associated with the moisture distribution patterns of the East Asian monsoon. Under conditions that meet transpiration demands, the seasonal precipitation pattern in East Asia plays a crucial role in mitigating high‐temperature stress through stomatal conductance regulation. Sufficient summer rainfall helps maintain leaf water potential and enhances the plant's ability to cope with extreme high‐temperature conditions (Wang et al. 2026).

Silt content (12.5%) suggests that soil with a moderate proportion of silt can minimize the risk of root hypoxia caused by precipitation fluctuations while providing sufficient aeration for root respiration. Studies have shown that, compared to sandy soils, plants of the same genus, *Acanthopanax senticosus* (Rupr. ex Maxim.) Harms., exhibit higher fine root biomass in soils with a silt content of 10%–15% (Hao et al. 2023), indicating that this soil structure can promote root proliferation and enhance nutrient uptake efficiency. The results of this study indicate that the suitable temperature range for A. sessiliflorus during the hottest month is between 20.05°C and 31.60°C, suggesting that this species has a certain level of adaptability to high temperatures. Compared with previous literature, which reported an optimal growth temperature of 22°C–25°C for A. sessiliflorus (Tong et al. 2015), there is some overlap between the two temperature ranges, both falling within the category of warm climate conditions. However, the temperature range obtained in this study is relatively broader, and the maximum value is significantly higher than previously reported. This suggests that, when selecting planting areas or introducing germplasm resources, it is necessary to comprehensively assess the species' suitability by considering specific environmental conditions and germplasm variations.

Under future climate change scenarios (SSP245), the weakening of the East Asian summer monsoon may lead to a 15%–20% reduction in precipitation in July–August across the study area, while the number of extreme high‐temperature days (> 30°C) is expected to increase by 5–8 days per year (Ding et al. 2024). These changes may pose challenges for northern and northeastern China, leading to reduced precipitation and increased extreme heat events (Wang, Fu, et al. 2022), thereby further reducing the suitable habitat for *A. sessiliflorus* .

### 4.2 Changes in the Suitable Habitat of *A. sessiliflorus* Under Current and Future Climate Change

As a typical woody plant in the temperate monsoon region, *A. sessiliflorus* exhibits sensitivity to climate change, revealing the dynamic coupling mechanism between species' ecological niches and critical climatic factors. This study constructed a three‐dimensional “area‐mass‐space” response model and found that the threshold effect of Max Temperature of Warmest Month (bio5) and the co‐evolution of species adaptation strategies constitute the core driving force. Under the high‐emission scenario SSP585, the area of high‐suitability habitat sharply decreases by 79.27%, which is closely related to the exceedance of the 35°C threshold for Max Temperature of Warmest Month and the resulting photosynthetic inhibition. The Sixth Assessment Report of the IPCC indicates that under high‐emission scenarios, the maximum temperature of the warmest month in temperate East Asia is expected to increase by 3.5°C–5.5°C (Intergovernmental Panel On Climate Change [IPCC] 2023), aligning closely with the spatiotemporal pattern of habitat collapse observed in this study. Mechanistic studies have shown that within the normal temperature range of 16°C–34°C, the Φ_PSIImax_ of plants can be stably maintained at a level of around 0.8. However, when the temperature exceeds its heat tolerance threshold, the Φ_PSIImax_ will decrease significantly (Neri et al. 2024). For C3 plants, high‐temperature‐induced non‐stomatal limitations dominate; for instance, *Acer platanoides* exhibits a nonlinear decline in stomatal conductance under extreme heat, leading to the decoupling of the photosynthetic assimilation rate from the electron transport chain (Marchin et al. 2022).

The heterogeneous development of areas of medium and low‐suitability reflects the adaptive differentiation strategies of species in response to hydrothermal variability. Simulations under the SSP126 climate scenario suggest that the extent of moderate‐suitability habitats for some temperate tree species may expand, possibly due to increased precipitation during the warmest season, which alleviates soil moisture limitations (Gu et al. 2025). Additionally, climate‐driven spatial reorganization of habitat patches may enhance landscape connectivity, thereby improving dispersal efficiency through animal‐mediated seed dispersal (Landim et al. 2025). However, under the high‐emission scenario SSP585, the range of moderate‐suitability habitats for some temperate tree species may significantly shrink. The primary drivers are the increasing frequency of extreme heat events and seasonal imbalances in summer precipitation, which impose adaptive selection pressure on species with low water use efficiency (WUE) due to elevated carbon acquisition costs (Liang et al. 2025).

The reduced expansion rate of low‐suitability habitats in the late SSP370 scenario may reflect a trade‐off between dual stresses. Some montane ecosystems enhance water retention capacity through higher soil organic matter content (Zhao et al. 2025), but rising temperatures during the warmest month increase evapotranspiration demand, exacerbating deep soil drying (Wang, Li, et al. 2022).

The spatial differentiation of centroid migration trajectories highlights the impact of topography on the redistribution of hydrothermal conditions. Under the SSP126 scenario, the centroid shift toward Chengde City is associated with changes in precipitation seasonality (bio18), thereby regulating local hydrothermal supply. Meanwhile, the relatively suitable climate in the northwestern inland region, characterized by smaller fluctuations in extreme temperatures, provides a buffer zone for the species' continued survival (Zhao, Zhang, et al. 2024). Under the SSP585 scenario, the unidirectional migration of the centroid toward Zhangjiakou City reflects the intensifying heat stress across the North China Plain, where the annual number of extreme heat days exceeds 15 (Ren et al. 2025). This pattern confirms that rising maximum temperatures (bio5) are forcing species to retreat to higher‐altitude refuges, reinforcing the critical role of topography in shaping species redistribution under climate change (Chen et al. 2024; Zhao et al. 2023).

Comparative analysis of suitable habitat under multiple climate change scenarios highlights the significant impact of mitigation measures on habitat conservation. Under the SSP126 scenario, the suitable habitat increases by 29.74%, indicating that emission reductions can mitigate habitat fragmentation and biodiversity loss (Song et al. 2025). However, under moderate‐emission scenarios, habitat quality declines by 23%–41%, suggesting that ecosystem degradation follows a nonlinear trend, necessitating adaptive management strategies (Xu 2025). In extreme scenarios, both habitat quantity and quality decline, demonstrating the cascading effects of climate tipping points on ecosystem services (Wunderling et al. 2024).

### 4.3 Potential Limitations

This study systematically revealed the critical environmental driving factors that affect the ecological suitability of *A. sessiliflorus* . However, it should be noted that there are still two technical bottlenecks to be overcome in the current research framework. First, constraints related to data availability and model operation preclude the development of a real‐time, dynamic prediction system capable of continuously updating habitat suitability in response to changing environmental conditions. Second, at the model algorithm level, only the MaxEnt model is used for spatial simulation, which limits the ability to capture the complementary strengths and uncertain benefits of multi‐model fusion. Future research should aim to overcome these limitations by building an integrated learning framework based on the stacking strategy and integrating the species interaction network model and the human activity intensity gradient model to achieve a multi‐dimensional coupling analysis of ecological niches and disturbance factors. However, it is worth emphasizing that this study has successfully achieved a high‐precision spatial simulation of the suitable habitat of *A. sessiliflorus* in China through the parameter‐optimized MaxEnt model, which can provide a reliable quantitative decision‐making basis for the in situ and ex situ conservation of this species.

## 5 Conclusion

This study systematically assessed the current and future potential suitable habitat distribution and evolutionary trends of *A. sessiliflorus* in China by integrating species distribution data with multiple environmental factors based on the optimized Maximum Entropy (MaxEnt) model. The model's training and testing AUC values were both above 0.96, indicating a high predictive accuracy. The results identified Temperature Seasonality (bio4), precipitation of Warmest Quarter (bio18), silt content, and Max Temperature of Warmest Month (bio5) as the key factors influencing the distribution of *A. sessiliflorus* . The response curves of these factors provide important insights into the ecological adaptability of this species.

Under different Shared Socioeconomic Pathway (SSP) scenarios, significant changes in the suitable habitat of *A. sessiliflorus* were observed. Under the low‐emission scenario (SSP126), moderate‐suitability and low‐suitability habitat exhibited a certain degree of expansion, providing a buffer space for the species. In contrast, under high‐emission scenarios (such as SSP585), the area of high‐suitability habitats sharply declined, and the overall suitable habitat showed a degradation trend, accompanied by a centroid shift toward the northwestern inland regions. This change not only reflects the direct impact of climate warming on the ecological niche of the species but also reveals the complexity of climate‐driven spatial reorganization of species distributions.

Despite the robust predictive performance of the model, certain limitations should be acknowledged. The analysis primarily incorporated environmental factors such as climate, soil, and topography, while biotic factors, including species interactions and human activities, were not incorporated. Additionally, the use of a single model somewhat constrained the comprehensiveness of the predictions. Future research should integrate multiple modeling approaches and incorporate factors such as biotic interactions and anthropogenic disturbances to further enhance predictive accuracy and practical application.

Overall, this study provides a scientific basis for understanding the spatial distribution and dynamic evolution of *A. sessiliflorus* suitable habitats under climate change. The findings have important implications for biodiversity conservation and regional ecological security.

## Author Contributions

**Yang Nan:** writing – original draft (equal). **Shuoning Zhang:** data curation (equal), writing – original draft (equal). **Wenjing Xu:** writing – review and editing (equal). **Kaize Feng:** writing – review and editing (equal). **Ziyang Liu:** visualization (equal). **Qingfeng Wang:** supervision (equal). **Xu Fan:** funding acquisition (equal).

## Funding

This work was supported by grants from the National Natural Science Foundation of China (No. 82204562), the Project of Construction of the Key Laboratory for Integrative Medicine Research on Diabetes in Liaoning Province (No. 2100224310), Basic Research Project of Liaoning Provincial Education Department (LJKMZ20221307), and Project of National Administration of Traditional Chinese Medicine (GZY‐KJS‐2022‐024).

## Conflicts of Interest

The authors declare no conflicts of interest.

## Supporting information


**Figure S1:** Correlation matrix of the 32 environmental variables.
**Figure S2:** Jackknife test of environmental variables in the MaxEnt model using all variables: (A) Jackknife of tarining gain for *A. sessiliflorus* ; (B) Jackknife of test gain for *A. sessiliflorus* ; (C) Jackknife of AUC for *A. sessiliflorus* .
**Table S1:** The contribution ratio of all environmental factors and the cumulative contribution ratio.

## Data Availability

All the required data are uploaded as Supporting Information.
