# Supplementary material for: Predicting Potential Distribution of the Acanthopanax Sessiliflorus in China Under Future Climate Scenarios Based the Optimized Maximum Entropy (MaxEnt) Model
Source: Ecol Evol. 2026 Jun 10;16(6):e73830. doi: 10.1002/ece3.73830 (PMC13250633; doi:10.1002/ece3.73830)
Supplement: Supplementary file 1 — Figure S1: Correlation matrix of the 32 environmental variables. Figure S2: Jackknife test of environmental variables in the MaxEnt model using all variables: (A) Jackknife of tarining gain for A. sessiliflorus ; (B) Jackknife of test gain for A. sessiliflorus ; (C) Jackknife of AUC for A. sessiliflorus . Table S1: The contribution ratio of all environmental factors and the cumulative contribution ratio. [file ECE3-16-e73830-s001.docx]

**Table S1** The contribution ratio of all environmental factors and the cumulative contribution ratio.

| **Variable** | **Percent contribution** | **Permutation importance** |
| --- | --- | --- |
| slope | 39.9 | 0 |
| bio4 | 23.1 | 57.1 |
| bio13 | 16.4 | 1.9 |
| cn_ratio | 4.9 | 9.6 |
| bio11 | 4.1 | 2.5 |
| bio18 | 2.4 | 3.3 |
| ph_water | 2.2 | 1.9 |
| elevation | 1.9 | 1.3 |
| silt | 1.8 | 0.4 |
| clay | 0.8 | 3.3 |
| bio12 | 0.7 | 14.1 |
| bio10 | 0.3 | 1.7 |
| sand | 0.3 | 0.1 |
| teb | 0.2 | 0.3 |
| bio16 | 0.2 | 0.4 |
| bio14 | 0.2 | 0 |
| bio7 | 0.1 | 0.2 |
| ref_bulk | 0.1 | 0.1 |
| coarse | 0.1 | 0.4 |
| bio2 | 0.1 | 0.2 |
| bio17 | 0 | 0.3 |
| bio9 | 0 | 0.3 |
| bio6 | 0 | 0.2 |
| bio3 | 0 | 0 |
| aspect | 0 | 0 |
| org_carbon | 0 | 0.1 |
| bio15 | 0 | 0 |
| bio1 | 0 | 0.1 |
| total_n | 0 | 0 |
| bio5 | 0 | 0 |
| bio8 | 0 | 0 |
| bio19 | 0 | 0 |

| **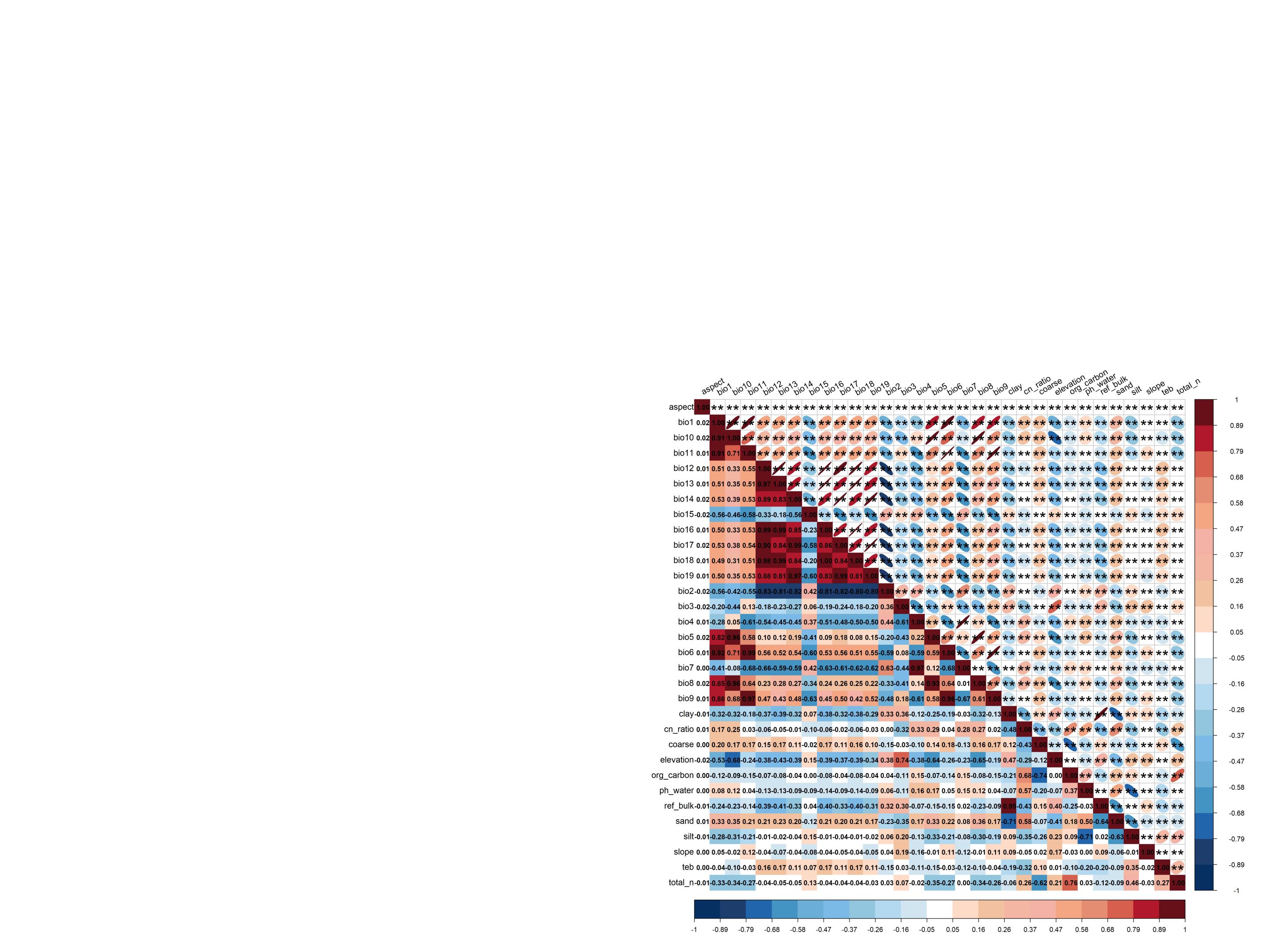** |
| --- |
| **Figure S1.** Correlation matrix of the 32 environmental variables. * denotes p < 0.05.** denotes p < 0.01. |

| **** |
| --- |
| **** |
| **** |
| **Figure S2** Jackknife test of environmental variables in the MaxEnt model using all variables: A) Jackknife of tarining gain for A. sessiliflorus; B) Jackknife of test gain for A. sessiliflorus ;C) Jackknife of AUC for A. sessiliflorus. |
